# Understanding Free Volume Characteristics of Ethylene-Propylene-Diene Monomer (EPDM) through Molecular Dynamics Simulations

**DOI:** 10.3390/ma12040612

**Published:** 2019-02-18

**Authors:** Yajian Wang, Yuyou Yang, Mingjiang Tao

**Affiliations:** 1 School of Engineering and Technology, China University of Geosciences (Beijing), 29 Xueyuan Road, Haidian District, Beijing 100083, China; yaron9@live.com; 2 Department of Civil and Environmental Engineering, Worcester Polytechnic Institute, Worcester, MA 01609, USA; taomj@wpi.edu

**Keywords:** EPDM rubber, molecular dynamics simulation, time temperature superposition principle, free volume theory

## Abstract

Understanding the underlying processes associated with the viscoelasticity performance of ethylene-propylene-diene monomer (EPDM) during its service life is essential for assessing and predicting its waterproofing performance in underground infrastructure. The viscoelasticity of the polymer is closely related to its free volume, and both of these properties depend on multiple factors, such as temperature, stress magnitude, and strain level. To explore the fundamental viscoelastic behavior of EPDM using free volume as a proxy for viscoelasticity, this article investigates the influence of temperature, stress magnitude, and strain level, as well as their combined effect, on the free volume through molecular dynamics (MD) simulations. An EPDM cross-linked molecular model was built and verified by comparing the simulation values of glass transition temperature, mechanical properties, and gas diffusivity with the experimental results reported in the literature. Then, the dependence of EPDM’s fractional free volume on temperature, strain, and their combined effect was investigated via MD simulations, on the basis of which the applicability of various superposition principles was also evaluated.

## 1. Introduction

Ethylene-propylene-diene monomer (EPDM) rubber—a copolymer with excellent resistance to oxidation, thermal stress, aging, and low permanent deformation—has been used in a wide range of industrial applications, such as waterproof joint sealing materials used for subway tunnels, pipe galleries, and underpasses, garden hoses, washers, roofing membranes, electrical insulation, and so on [1,2,3,4]. As a waterproofing seal in underground infrastructure, EPDM’s viscoelasticity plays an important role for its long-term performance. Throughout its service life, EPDM is subjected to the combined effect of various mechanical and environmental loading, which often alters its viscoelasticity and leads to the deterioration of its performance. For example, the hardening of EPDM makes it less flexible and more prone to cracking and thus results in water leakage [5,6]. Therefore, it is important to understand the underlying processes associated with EPDM’s viscoelasticity in order to assess and predict its waterproofing performance in underground infrastructure. Wu et al. (2008) [7], Zeng et al. (2011) [8], and C Shi et al. (2015) [9] conducted accelerated aging tests on EPDM on the basis of the Arrhenius equation to explore its long-term performance. Similar to other viscoelastic materials, EPDM’s behavior and performance as a waterproofing seal in underground infrastructure depend on multiple factors, such as temperature, loading rate (time), strain level, stress (pressure) magnitude, and their combined effects. The equivalency of time and temperature [10] and the superposition of temperature and stress [11] were experimentally investigated and exploited to characterize the long-term performance of polymers. Markovic et al. (2000) [12] studied the viscoelastic behavior of EPDM elastomer and reported that, in short-time testing, EPDM follows time–temperature equivalence. S Ronan et al. (2007) [13] predicted the long-term stress relaxation of EPDM elastomers using the time–temperature superposition method. T Cui et al. (2012) [3] studied the stress relaxation behavior of EPDM seals in a fuel cell environment using time–temperature superposition. Wang et al. (2017) [14] investigated the strain–time performance of the fiber material using the time–stress superposition principle. Time–temperature equivalence, and sometimes temperature–stress or temperature–strain equivalence, has been applied by using a shifting factor to predict EPDM’s viscoelasticity over much longer time periods or larger stress or strain magnitudes than laboratory tests would allow [15]; however, few studies have been devoted to validating the above equivalences, nor have their microscopic mechanisms been revealed. Therefore, this study aims to reveal microscopic aspects associated with the viscosity of EPDM and their dependence on temperature, time, and the magnitudes of stress and strain through molecular dynamics (MD) simulations. Several MD simulations of the transport properties of polymers have been reported in the literature. Wu et al. (2006, 2007) [16,17] constructed a cross-linked model of epoxy resin and investigated the diffusion behavior of the water in the molecule. Rutherford et al. (2007) [18] used the MD and Grand Canonical Monte Carlo (GCMC) modules to monitor the diffusion and dissolution of helium, hydrogen, oxygen, and other gases in EPDM. In addition, MD simulations were used to model the cross-linking process of the polymer. Varshney et al. (2008) [19] proposed a multistep relaxation procedure for relaxing the molecular topology during cross-linking process. Maly et al. (2008) [20] simulated the (3-glycidoxypropyl) trimethoxy silane (GPTMS) cross-linking reaction using molecular dynamics and the assumption that close proximity was a criterion for the occurrence of cross-linking. Aghadavoudi (2017) [21] investigated the effects of resin cross-linking ratio on the mechanical properties of epoxy-based nanocomposites using molecular dynamics.

Although the viscoelasticity of EPDM is the property directly related to its long-term performance as a waterproofing seal and thus the interest of the study, free volume was chosen as a proxy for viscoelasticity for the following reasons: (i.) as an intrinsic property of the polymer, free volume controls its molecular mobility and reflects the inherent timescale and thus viscosity of the materials [11,22,23,24] (e.g., the free volume concept provides the theoretical basis for the Williams–Landel–Ferry (WLF) equation for time–temperature superposition); and (ii.) MD simulations of the viscoelasticity of EPDM is more challenging in practice due to much higher computational costs than simulations of free volume. Free volume is an intrinsic defect of polymer materials, including atomic-scale and molecule-scale holes between the molecular chains and the structural defects caused by random packing of the molecular chains.

In this study, MD simulation was used to study the free volume characteristics of EPDM, particularly its dependence on temperature, stress, and strain, as well as the feasibility of applying the superposition among the above factors in order to understand and predict the long-term viscoelastic behavior of EPDM. The article is organized according to the following steps implemented in the study: (1) an MD model for cross-linked EPDM was built by writing a Perl script developed in Materials Studio v. 8.0; (2) the built MD model of EPDM was first verified by comparing it with EPDM’s experimentally determined thermal (glass transition temperature), mechanical (the stress–strain relationship), and transport (the gas diffusion coefficients) properties reported in the literature; and (3) the validated MD model was used to further investigate the free volume characteristics of EPDM, with a focus on the influence of temperature, stress magnitude, strain level, and their combined effect, as well as any equivalent effect among these influencing factors.

## 2. MD Simulation Methods

MD simulations were carried out to examine the behavior of the free volume of EPDM polymer in response to temperature, stress magnitude, and strain level; these observations provide microscopic insights into whether various superposition principles are applicable for predicting long-term viscoelastic behavior of EPDM as a waterproofing seal in underground infrastructure. An EPDM MD model was first built with Perl scripts with consideration of its cross-linking, and the model was verified using available experimental results reported in the literature. Then, additional MD simulations were performed on the validated MD model to explore its free volume and its dependence on temperature, stress, and strain.

### 2.1. Molecular Dynamics Simulations

#### 2.1.1. Potentials

COMPASS (Condensed-phase Optimized Molecular Potentials for Atomistic Simulation Studies) was used to simulate EPDM’s structural and thermophysical properties, since it has been parameterized to predict various properties and successfully applied in the MD simulations of polymers [18,21,25]. COMPASS is an ab initio forcefield that was validated using condensed-phase properties, in addition to various ab initio and empirical data for molecules in isolation. Consequently, this forcefield enables the accurate and simultaneous prediction of structural, conformational, vibrational, and thermophysical properties for a broad range of polymeric molecules in isolation and in condensed phases under a wide range of temperature and pressure conditions.

With the consolidated coverage of inorganic and organic materials in the COMPASS forcefield, the nonbonded functional forms used for different materials are consistent with the framework of COMPASS parameters so that the nonbonded parameters are generally transferable. Therefore, interactions between different molecular systems can be modeled with the assumption that only nonbonded interactions exist between two systems of interest. A simple ionic model [26] that consists of the Coulombic term and the vdW term is used for ionic species in COMPASS. In this model, every atom is treated as a nonbonded particle—there is no valence bonding between any pair of atoms. Mathematically, the ionic bond is represented by the strong attractive force between oppositely charged atoms and the vdW terms whose repulsion part keeps the oppositely charged atoms at a certain distance from each other.

(1) $$E_{pot}=\sum_{i>1}\frac{q_{i}q_{j}}{r_{ij}}+\sum_{i>j}E_{ij}\left[2{\left(\frac{r_{ij}^{0}}{r_{ij}}\right)}^{9}-3{\left(\frac{r_{ij}^{0}}{r_{ij}}\right)}^{6}\right]$$

where *r_ij_* is the distance between ions *i* and *j*, and $r_{ij}^{0}$ and *E_ij_* are the corresponding vdW parameters for the *ij* ion pair:

(2) $$r_{ij}^{0}={\left(\frac{{\left(r_{i}^{0}\right)}^{6}+{\left(r_{j}^{0}\right)}^{6}}{2}\right)}^{\frac{1}{6}}$$

(3) $$E_{ij}=2{\left(E_{i}E_{j}\right)}^{\frac{1}{2}}\frac{{\left(r_{i}^{0}\right)}^{3}{\left(r_{j}^{0}\right)}^{3}}{{\left(r_{i}^{0}\right)}^{6}+{\left(r_{j}^{0}\right)}^{6}}$$

The charges *q_i_*, *q_j_* are electron charges which, in most cases, are equivalent to oxidation states. A modified bond increment charging scheme is used for their calculation:

(4) $$q_{i}=\delta_{i}+\sum \delta_{ij}$$

For ionic materials, *δ_i_* is set to the formal charge (±1); otherwise, it is zero, and *δ_ij_* is zero for ionic materials.

#### 2.1.2. Creating a Cross-Linked MD Model

**Figure 1:**
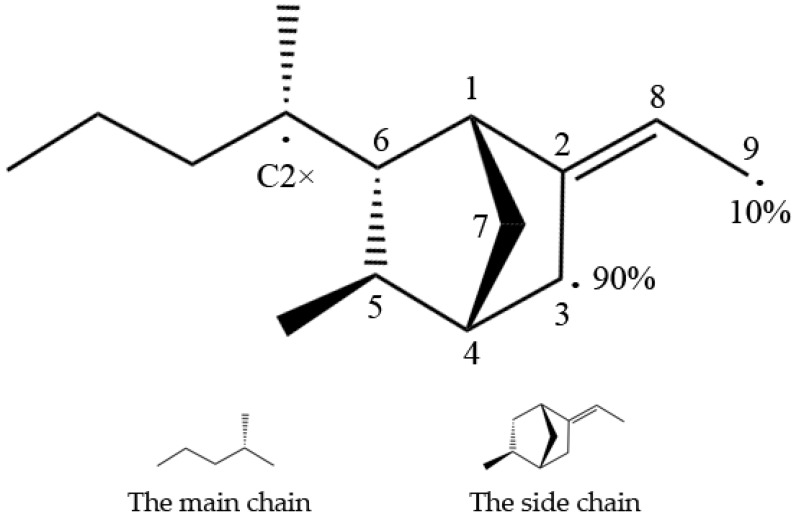
The active positions of ethylene-propylene-diene monomer (EPDM) free groups. The active position on the main chain is C2×, and the active positions on the side chain are C3 and C9. The reaction rate of C3 in the monomer is 90%, while C9 is only 10%.

Understanding the chemical process of the EPDM’s cross-linking reaction is necessary to create a cross-linked EPDM molecular structure. The cross-linking process of EPDM with a third monomer of 5-ethylidene-2-norbornene (ENB), which is commonly added to a sealing EPDM gasket, is briefly explained here. Previous studies have shown that peroxide free radicals mainly capture the tertiary hydrogen (C2×) on the main chain and the hydrogens at the C3 and C9 positions on ENB; the locations are schematically shown in Figure 1 [27]. For actual EPDM, the ratio of the main chain to the side chain is about 9:1. In addition, Mouna (2008) [28] showed that the reaction rate of C3 in the monomer was 90%, while that of C9 was only 10%. Therefore, the reaction with atom C9 was omitted because its quantity and reaction rate are quite low. The atom C2× of the main chain and the atom C3 of the side chain were identified as the target cross-linked atoms.

**Figure 2:**
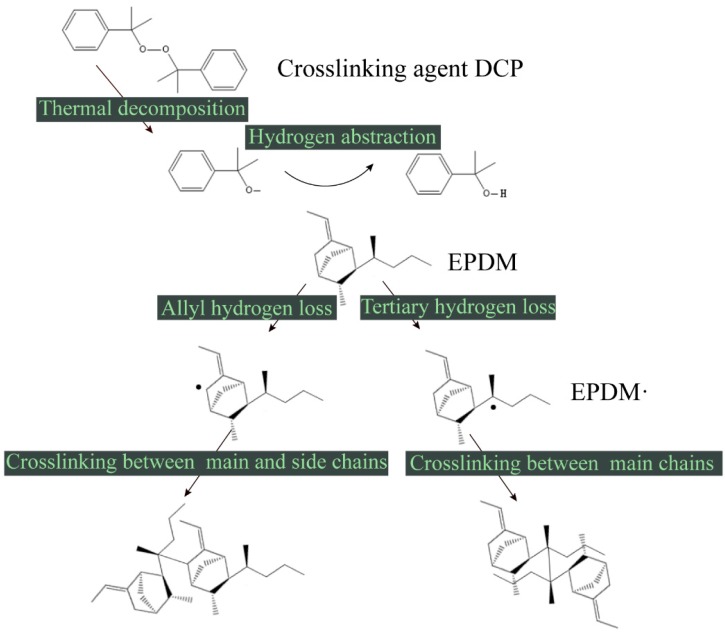
Theoretical cross-linking process of EPDM by dicumyl peroxide (DCP).

The cross-linking process of EPDM is schematically shown in Figure 2. During the depicted process, peroxides undergo thermal decomposition to produce the alkoxyl radical RO**·**, which grabs the H atoms on the EPDM main chain and the unsaturated third monomer to produce the macromolecular free radical EPDM**·**.

**Figure 3:**
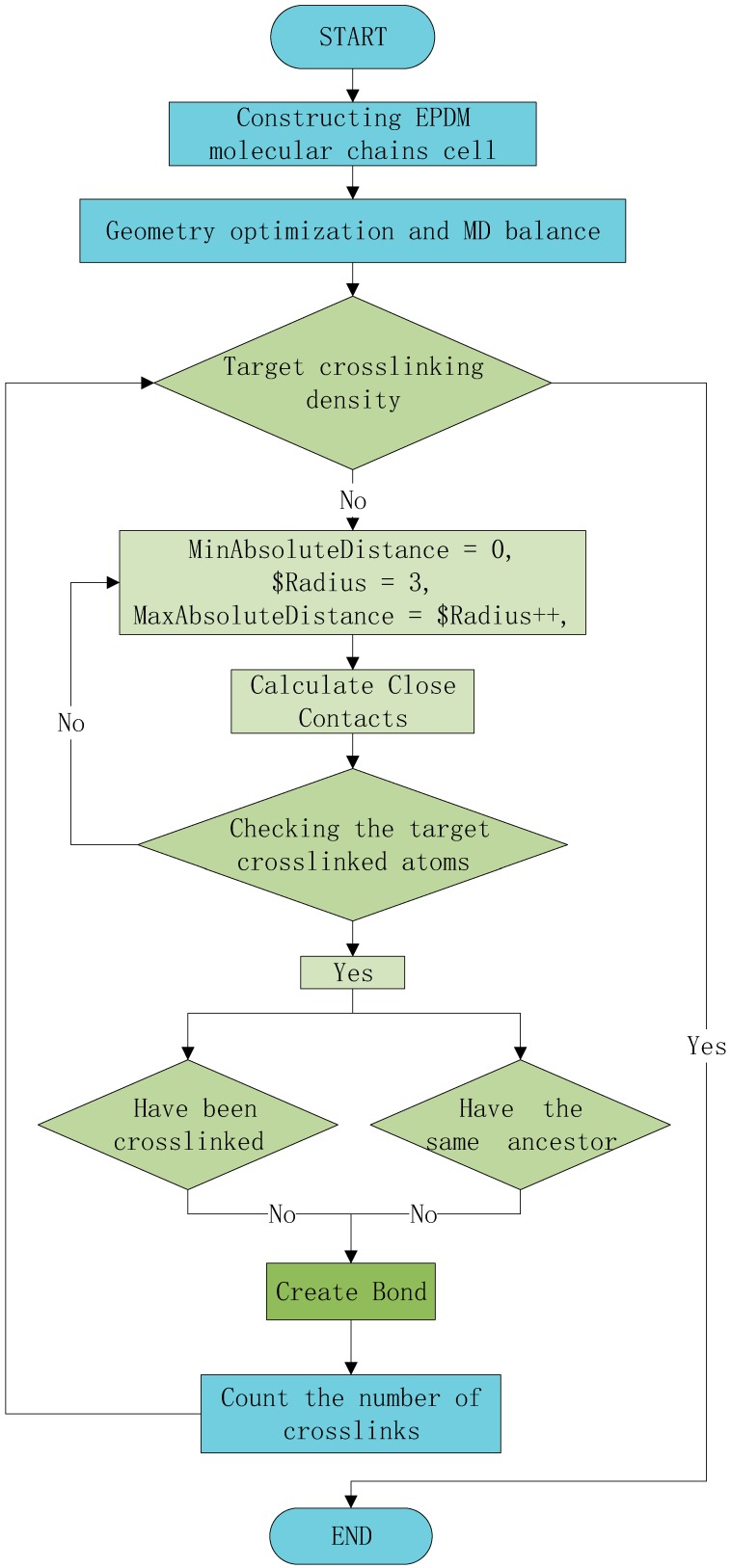
The cross-linking loop algorithm.

According to the actual production ratio, ethylene [29,30], propylene, and the third monomer (ENB) were copolymerized in a ratio of 5:4:1 to form an EPDM molecular chain. Then, 10 molecules were randomly added to an amorphous periodic cell at a temperature of 298 K, density of 0.87 g/cm^3^, and length of 29.08 Å for cross-linking. This was followed by minimizing the potential energy of the polymer system. The whole cross-linking process was completed in the amorphous periodic cell and was achieved by Perl script. The process of modeling the cross-linking of EPDM during MD simulations is shown in Figure 3, with the key aspects of modeling the cross-linking process listed below.

The algorithm of several key steps for simulating the EPDM cross-linking process is as follows:

Step 1 The system was equilibrated twice using NPT for 20 ps and NVT for 20 ps, respectively.

Step 2 Closecontacts were calculated. According to our case, the MinAbsoluteDistance and the MaxAbsoluteDistance were set as 0 and 3 Å, respectively. The cross-linking density was used as a reference for the beginning of the next loop, which increases the MaxAbsoluteDistance, until the target cross-link number was obtained.

Step 3 Cross-link bonds were created. Closecontacts for any combination of C2X and C3 were identified, and then the contacted atoms that do not belong to the same molecule were screened out to create bonds and placed in an array.

Step 4 Atoms that had been cross-linked were not computed in the next time step. The grep function, which can screen out atoms that already exist in the array, was used to avoid the repeated bonding of atoms.

Step 5 Ultimately, counting the array elements returned the value of the cross-link density.

**Figure 4:**
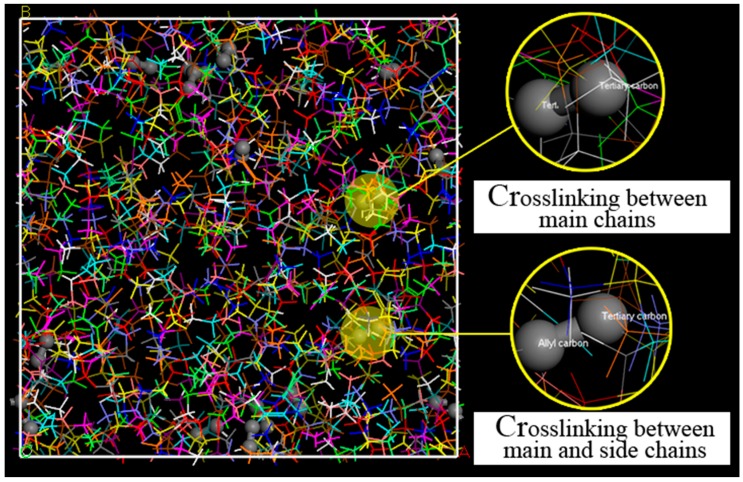
EPDM cross-linking structure, where the C–C bonds generated are displayed as balls. The top structure, enlarged from the yellow area, represents the main chains cross-linking with each other; the bottom structure is the cross-link between the main chain and side chain.

To get a stable enough system, the cross-linked EPDM cells were annealed through temperature cycles of ramping up and down at a rate of 5 K/step, with an initial temperature of 150 K and a mid-cycle temperature of 800 K. The cross-linked molecular EPDM model is shown in Figure 4.

### 2.2. Verification of the MD Model

The experimental results of the glass transition temperature, mechanical properties, and transport properties of EPDM reported in the literatures were used to validate the EPDM model that was created as described in the preceding section. This was achieved by a series of NPT MD simulations at temperatures of 148, 178, 208, 238, 268, 298, 333, 363, 393, and 423 K and under stress magnitudes of 2, 4, 6, 8, 10, 12, and 14 GPa, with the aid of a Berendsen thermostat and a Souza-Martins barostat for 100 ps.

#### 2.2.1. Glass Transition Temperature

**Figure 5:**
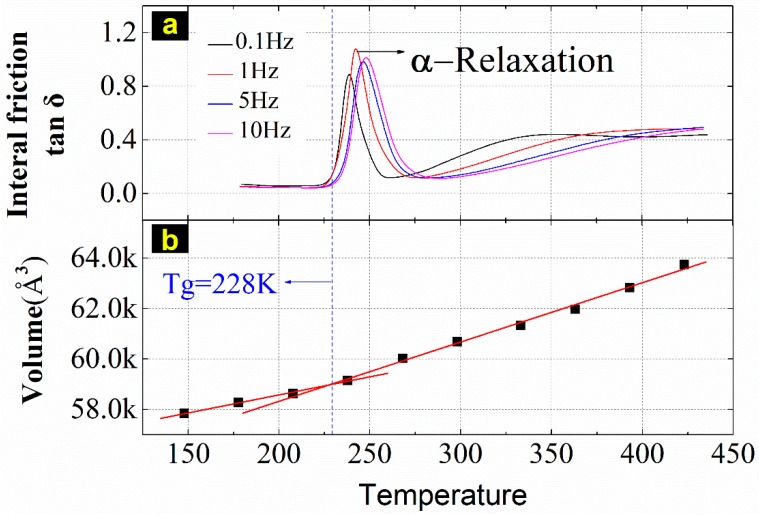
The glass transition temperature of EPDM: (**a**) shows the relaxation curve of 0.1, 1, 5, and 10 Hz at temperatures ranging from 183 to 438 K. *δ* is the phase angle. The simulation results in (**b**) are the average of three independent NPT simulations for 20 ps under the compass field. The volumes at temperatures of 148, 178, 208, 238, 268, 298, 333, 363, 393 and 423 K are 57,853.14, 58,284.27, 58,645.86, 59,164.04, 60,023.98, 60,683.87, 61,327.6, 61,969.22, 62,836.05, and 63,743.02 Å^3^, respectively.

The glass transition temperature can be obtained by fitting the EPDM cell volume at different temperatures. In Figure 5, the upper part is the mechanical relaxation spectrum of EPDM, in which the first curvilinear peak is called the α relaxation, which is thought to be caused by the glass transition, while the lower part is the linear volume fitting at different temperatures. Therefore, the MD simulations indicate that the glass transition should occur at around 228 K, (i.e., the temperature at which the relaxation occurs), which agrees well with what Gu et al. [31] reported, −43 °C (230 K).

#### 2.2.2. Gas Diffusivity

**Figure 6:**
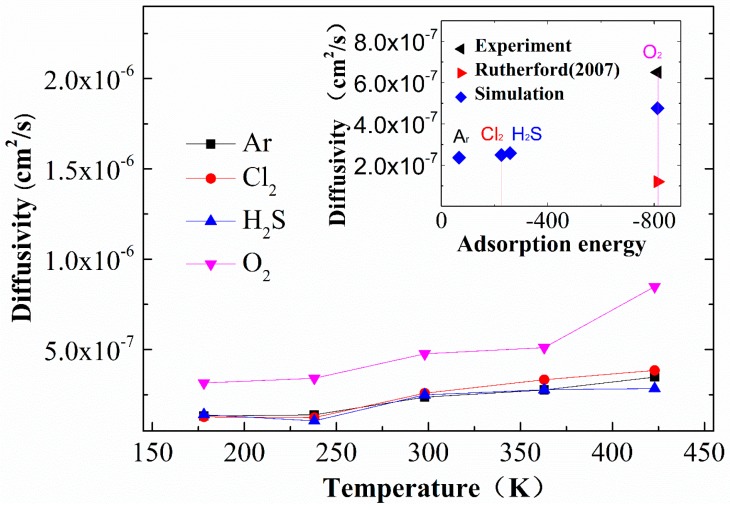
The diffusion coefficients of oxygen, hydrogen sulfide, argon, and chlorine at different temperatures are represented by colored curves. The inset shows the comparison between the results of this study’s simulation of oxygen and those reported by Rutherford [18].

Although EPDM is a saturated rubber with strong chemical stability, it will be degraded by some chemical gases during long-term service, and the degradation is affected by the diffusivity of these gases in EPDM. As described in this section, the diffusion of O_2_, H_2_S, Cl_2_, and Ar in EPDM was investigated with the knowledge that the transport behavior of these gases has been previously reported to degrade EPDM. As expected, as the temperature increases, the gas diffusivity increases for all of the simulated gases (see Figure 6). There are two mechanisms underlying the increasing gas diffusion: one is the free volume expanding with rising temperature, thus providing more space for the diffusion of gas, and the other is higher temperatures accelerating the movement of gas and EPDM molecular segments, making it easier to form connected diffusion channels. As shown in the upper right inset, compared with the literature data, the simulation results of O_2_ diffusion are closer to experimental values than Rutherford’s simulation result [18], because the cross-linked EPDM model in the current study is closer to experimentally observations.

#### 2.2.3. Mechanical Property of EPDM

At constant temperature (canonical ensemble), the adiabatic elastic constants can be obtained at constant energy (microcanonical ensemble). The method has the advantage that all elastic constants can be obtained from a single simulation. The disadvantage, however, is that the simulation must be long enough to guarantee calculation accuracy. A more direct approach, which also takes into account entropic effects, is provided by non-equilibrium methods, in which the system is subjected to finite stress, and the resulting strain is measured. The Parrinello-Rahman or the Souza-Martins barostat can be used to apply constant pressure, allowing cell lengths and angles to fluctuate, from which strains can be derived [32].

**Figure 7:**
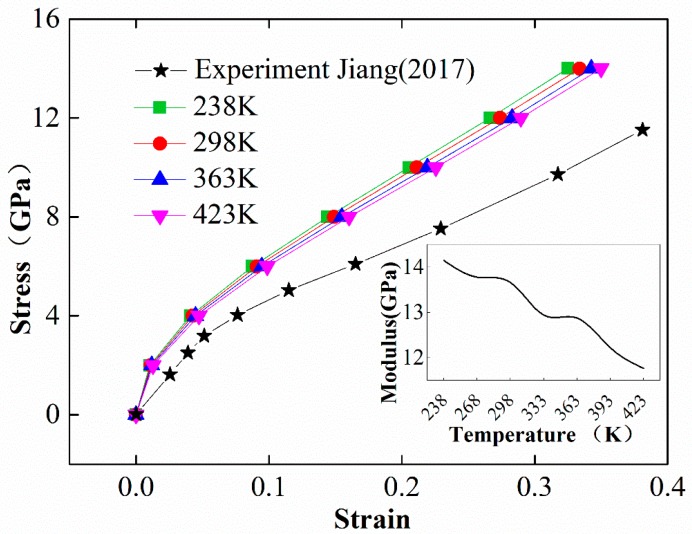
The colored curves are stress–strain curves of EPDM, the star curve is the uniaxial compression EPDM stress–strain curve measured by Jiang at 298 K, and the inset is the modulus (the ratio of stress to strain when the stress magnitude is equal to 14 GPa) at different temperatures.

The stress and strain curves of the EPDM model were obtained through a Souza-Martins barostat applying 1-d loading of 2, 4, 6, 8, 10, 12, and 14 GPa at temperatures of 238, 298, 363, and 423 K. The calculated results were compared with the experimental data of Jiang (2017) [33]. As shown in Figure 7, due to the difference in the scale between the MD simulation and the experiment by Jiang, the simulation results are much larger than the experimental counterparts. The star curve represents the experimental uniaxial compression test data with the unit MPa. As can be seen in the figure, the results of simulation and experiment are consistent qualitatively in terms of their trends. The modulus of EPDM depends on the level of strain, as the slope of the stress–strain curves decreases as the strain increases when the strain is less than 0.1, while it is almost constant when the strain is between 0.1 and 0.35. The simulated stress–strain curves also exhibit temperature dependence at the same load level: the strain increases with increasing temperature, and the larger the strain, the more significant the impact. This phenomenon is well known for viscoelastic materials, such as EPDM, and can be attributed to the weakened interactions between molecules by the accelerated motion of molecule segments at higher temperatures.

The inset of Figure 7 describes the ratio of stress to strain (modulus) when the stress magnitude is equal to 14 GPa at different temperatures, which shows that the derived modulus decreases in a stepwise pattern as the temperature rises due to the influence of the characteristic temperature. The colored curves were obtained using the Souza-Martins barostat at temperatures of 238, 298, 363, and 423 K.

### 2.3. Molecular Simulation of Free Volume

**Figure 8:**
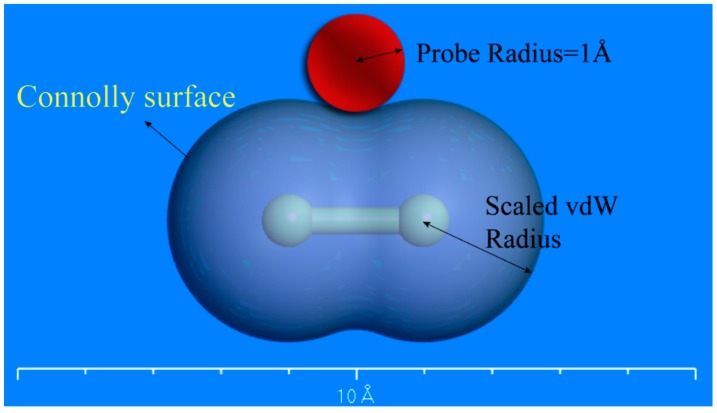
Connolly surface of a chlorine molecule.

Free volumes of simulated EPDM models are available using the “atom volumes and surfaces” functionality of the Materials Visualizer, which is calculated through probe scanning [34]. As shown in Figure 8, the boundary between the probe and the atoms is defined as the atom volume surface (Connolly surface). Free volume is the one on the side of the atom volume surface without atoms, and the occupied volume is the one on the atom side of the atom volume surface.

A series of EPDM MD models at different temperatures and different strain levels were obtained during the calculations of the glass transition temperature and stress–strain curves. The free volume of those models was calculated by creating an atom volume surface (Connolly surface). The periodic box was divided into several grids with an interval of 0.15 Å, and all grids were scanned by a probe with radius of 1 Å. If more than half of the space of the grid was occupied by a probe, it was marked as accessible. If more than half of the space of the grid was occupied by atoms, it was marked occupied. Free volume is formed when two unoccupied cells are arranged in adjacent positions; the ratio of the free volume to the total volume is the fractional free volume (FFV) [35].

**Figure 9:**
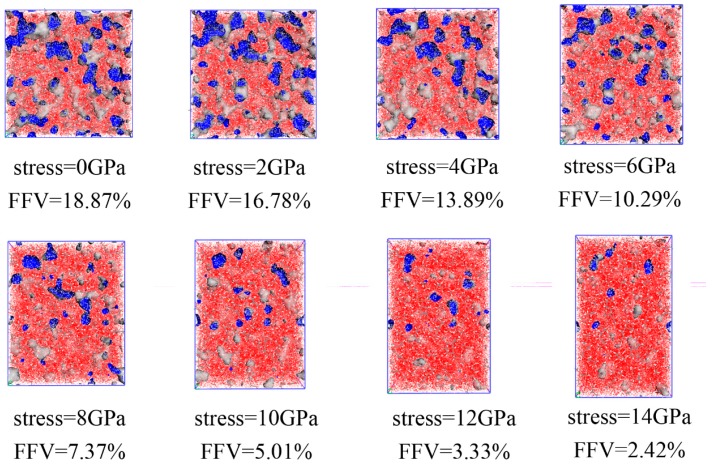
The volume change of EPDM during compression, showing the compression of EPDM from 0 to 14 successively. The blue area represents the surface of free volume through the pore channel, the white area is the pore space, the gray area is the skeleton of the pore channel, and the red area is the occupied volume.

The snapshot of free volume distribution of EPDM at different stress levels under a compression process is shown in Figure 9.

## 3. Results and Discussion

The FFV’s calculation results were used to understand the effects of key factors on the FFV of EPDM, with a focus on the influence of temperature, stress magnitude, strain level, and their combined effect, as well as any equivalent effect among these influencing factors. In this section, three principles to explain the superposition of time, temperature, and stress are discussed first. Superposition can describe the behavior of viscoelastic materials undergoing accelerated relaxation by high temperature and high loads.

### 3.1. Principles of Superposition between Time, Temperature and Strain

According to the free volume theory, the viscosity η changes with the free volume fraction *f*, and the relationship between them can be expressed by the Doolittle equation.

(5) $$\eta =A\exp\left[B\left(\frac{1}{f}-1\right)\right]$$

where *A* and *B* are constants related to materials.

The intrinsic time of viscoelastic materials is affected by temperature. A lot of research studies have shown that the relaxation of molecular motion takes longer at low temperatures than at high temperatures. Stress magnitude also affects the intrinsic time of viscoelastic materials. The same relaxation phenomenon of materials can be observed at short times with high stress levels and at long times with low stress levels. Considering that the relaxation characteristics of viscoelastic materials can be accelerated by high stress and high temperature, some scholars proposed the TTSP (time–temperature superposition principle), TSSP (time–stress superposition principle), and TTSSP (time–temperature–stress superposition principle).

According to TTSP, mechanical behaviors of viscoelastic materials at different temperatures can be linked by changing the time domain. The long-term viscoelastic diachronic curve of a reference temperature can be obtained by shifting the log-time coordinates of a series of curves at different temperatures in a short time. TTSP assumes that if the curve of the relaxed modulus *E* of EPDM at temperature *T* shifts log$\phi_{T}$ along the logarithmic time axis, it will coincide with the curve of *T*_0_. The relaxation modulus *E* has the following relation:

(6) $$E\left(T,t\right)=E\left(T_{0},t/\phi_{T}\right)$$

where *T* is the target temperature; *T*_0_ is the reference temperature; $\phi_{T}$ represents the temperature shift factor.

According to the TSSP, the relaxation modulus *E* has the following relation:

(7) $$E\left(\sigma ,t\right)=E\left(\sigma_{0},t/\phi_{\sigma }\right)$$

where σ is the target stress; $\sigma_{0}$ is the reference stress; $\phi_{\sigma }$ represents the stress transformation factor.

The time–temperature–stress superposition principle assumes that the free volume fraction is linearly related to the force and temperature. Therefore, the free volume fraction can be expressed as follows:

(8) $$f=f_{0}+\alpha_{T}\left(T-T_{0}\right)+\alpha_{\sigma }\left(\sigma -\sigma_{0}\right)$$

where $\alpha_{T}$ is the temperature expansion coefficient of the free volume fraction; $\alpha_{\sigma }$ is the stress expansion coefficient; $f_{0}$ is the reference free volume fraction.

Considering that the reduction of free volume caused by stress is mainly due to the compression of interstitial spaces in the structure, the stress in the time–temperature–stress superposition principle was replaced by strain in this study—namely, the time–temperature–strain superposition principle (TTSSP). Then, the free volume fraction equation can be expressed as follows:

(9) $$f=f_{0}+\alpha_{T}\left(T-T_{0}\right)+\alpha_{\varepsilon }\left(\varepsilon -\varepsilon_{0}\right)$$

where ε is the target strain; $\varepsilon_{0}$ is the reference strain; $\alpha_{\varepsilon }$ is the strain expansion coefficient.

According to Equations (8) and (9), the temperature expansion coefficient and strain expansion coefficient can be calculated by the free volume simulation results, and then the temperature shift factor, strain shift factor, and temperature strain shift factor can be obtained by the methods described below.

If $\phi_{T\varepsilon }$ represents the temperature–strain shift factor, the viscosity has the following relationship:

(10) $$\eta \left(T,\varepsilon \right)=\eta \left(T_{0},\varepsilon \right)\phi_{T}=\eta \left(T,\varepsilon_{0}\right)\phi_{\varepsilon }=\eta \left(T_{0},\varepsilon_{0}\right)\phi_{T\varepsilon }$$

Substitute Equation (10) into Equation (5):

(11) $$\mathrm{lg}\phi_{T}=\frac{-C_{1}\left(T-T_{0}\right)}{C_{2}+T-T_{0}}$$

(12) $$\mathrm{lg}\phi_{\varepsilon }=\frac{-C_{1}\left(\varepsilon -\varepsilon_{0}\right)}{C_{3}+\varepsilon -\varepsilon_{0}}$$

where $C_{1}$ = $B/{2.303f}_{0}$, $C_{2}$ = $f_{0}/\alpha_{T}$, $C_{3}$ = $f_{0}/\alpha_{\varepsilon }$.

The temperature shift factor ($\phi_{T}$) of the time–temperature superposition principle is expressed by Equation (11). The strain shift factor ($\phi_{\varepsilon }$) of the time–strain superposition principle (TSSP) is represented by Equation (12).

### 3.2. The Dependence of EPDM’s Free Volume on Temperature

**Figure 10:**
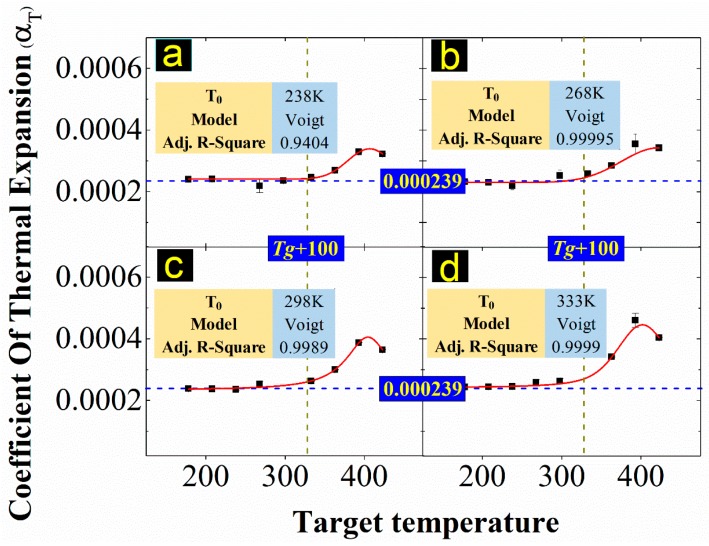
Thermal expansion coefficient: $T_{0}$ is the reference temperature and $T_{g}$ is the glass transition temperature; (**a**–**d**) show the coefficient of thermal expansion at reference temperatures of 238, 268, 298, and 333 K, respectively. The results were fitted by the Voigt model.

The dependence of EPDM’s free volume on temperature was examined on the basis of the thermal expansion coefficient. The well-known WLF equation of the TTSP (Equation (11)) is based on the assumption that when the temperature is in the range of $T_{g}$~$T_{g}$ + 100, the fractional free volume varies linearly with temperature. The simulation results of thermal expansion coefficients of EPDM at different reference temperatures are shown in Figure 10.

As shown in Figure 10, the variation trend of the thermal expansion coefficient is consistent with the four reference temperatures. When the target temperature is lower than 328 K (that is, equal to $T_{g}$ + 100), the thermal expansion coefficient remains nearly constant at 0.000239. When the target temperature exceeds 330 K, the thermal expansion coefficient, which is no longer a constant, increases to a peak and then falls back. The results conform to the applicable the WLF equation’s temperature range, $T_{g}$~$T_{g}$ + 100 ($T_{g}$ = 228 K is obtained in Figure 6).

**Figure 11:**
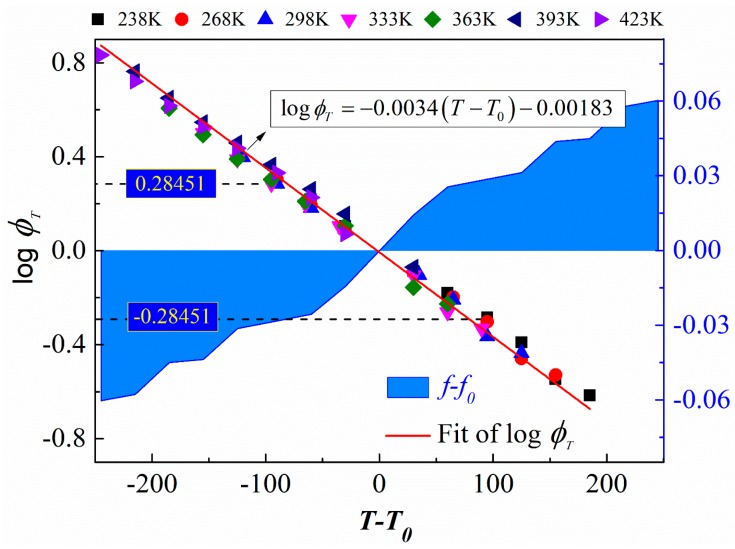
The dependence of the temperature shift factor and fractional free volume on temperature. Colored symbols indicate temperature shift factors at target temperatures of 238, 268, 298, 333, 363, 393, and 423 K. The red curve is the fit of the temperature shift factor. The blue areas are the increments in the fractional free volume with the temperature gradient.

The temperature shift factor is used to obtain long-term performance curves (master curve) of viscoelastic materials and can be calculated by Equation (11). According to the free volume simulations, the temperature shift factor was derived, and the results at different target temperatures are shown in Figure 11.

Figure 11 shows that the fit of the temperature shift factors decreases linearly as the temperature gradient rises, which means that the viscosity of EPDM also linearly changes with an increasing temperature gradient. The increase in temperature causes the free volume to increase and the absolute value of the transfer factor to increase, which indicates an increase in the time range characterizing long-term performance. When the target temperature is 333 K, the reference temperature is 238 K; the temperature shift factor is 0.28451, while the reverse is −0.28451. This phenomenon indicates that the effect of temperature on viscosity is reversible.

### 3.3. The Dependence of EPDM’s Free Volume on Load Magnitude

**Figure 12:**
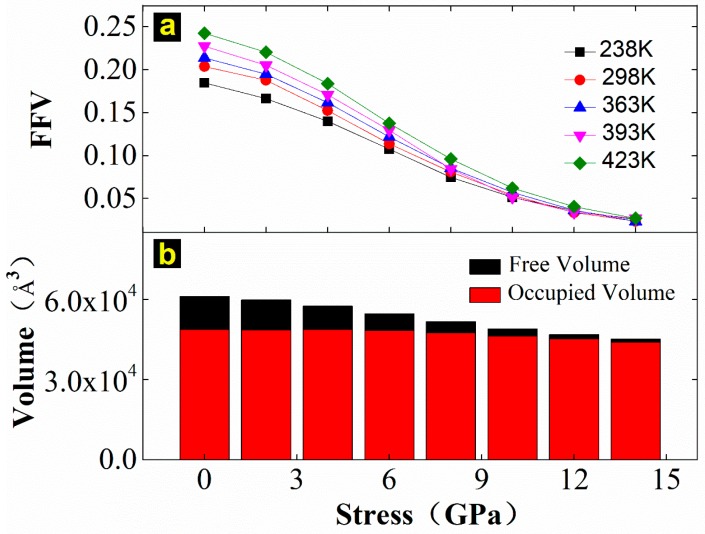
Influence of temperature and strain on the free volume of EPDM: (**a**) shows the variation curve of free volume with different compression stresses at different temperatures, (**b**) is the variation trend of the occupied and free volumes with varying stresses of compression at 298 K.

The calculation results of FFV under different stress magnitudes at temperatures of 238, 298, 363, 393, and 423 K are shown in Figure 12a. The FFV increases with increasing temperature and decreases with the increasing stress magnitude, and the effect of stress on the free volume fraction is more significant than that of temperature. It can be seen from Figure 12b that not only is the free volume reduced when the stress increases, but the occupied volume is also compressed when the stress is greater than 6 GPa.

**Figure 13:**
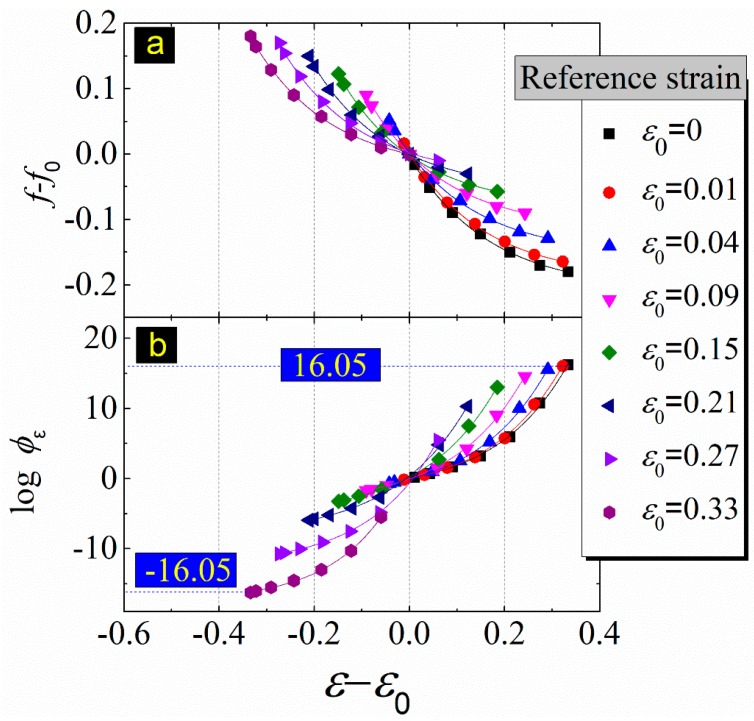
(**a**) Strain expansion coefficients (the slope of colored curves); (**b**) strain shift factors at reference strain of 0.01, 0.04, 0.09, 0.15, 0.21, 0.27, and 0.33.

The change in FFV caused by the stress magnitude mainly depends on the strain level. The strain expansion coefficient and the strain shift factor, which can be derived from Equations (9) and (12), were introduced to understand the dependence of fractional free volume of EPDM on the strain level. The relationship between fractional free volume and the strain magnitude and the strain expansion coefficient were derived from MD simulations and are shown in Figure 13.

It can be seen from Figure 13a that the fractional free volume decreases exponentially with increasing strain magnitude, which means that, unlike the thermal expansion coefficient, the strain expansion coefficient is nowhere near constant. This invalidates the assumptions of Equation (9). The relationship between the fractional free volume and the strain level can be expressed by a fitting formula:

(13) $$f-f_{0}=A\exp\left(-\frac{25\left(\varepsilon -\varepsilon_{0}\right)}{4}\right)+B$$

The strain expansion coefficient can be derived from the derivative of Equation (13).

(14) $$\alpha_{\varepsilon }=-\frac{25}{4}A\exp\left(-\frac{25}{4}\left(\varepsilon -\varepsilon_{0}\right)\right)$$

where $A\;\approx {\;f}_{0}$, $B\;\approx \;{-f}_{0}$. The goodness-of-fit test results of Equation (13) are shown in Table 1.

As shown in Figure 13b, the strain shift factor increases exponentially with the strain increase. For the effect of the strain magnitude on EPDM’s viscoelastic performance, the process of strain shifting from high to low and from low to high is reversible. When the reference strain is 0.01, and the target strain is 0.33, the calculated value of the strain shift factor is 16.05; on the contrary, the calculated value of the strain shift factor is −16.05. The stress shift factor is much larger than the temperature shift factor. It can be noticed that the time range for characterizing long-term performance of loads is longer than that of temperature.

### 3.4. The Feasibility of the Superposition between Temperature and Strain

**Figure 14:**
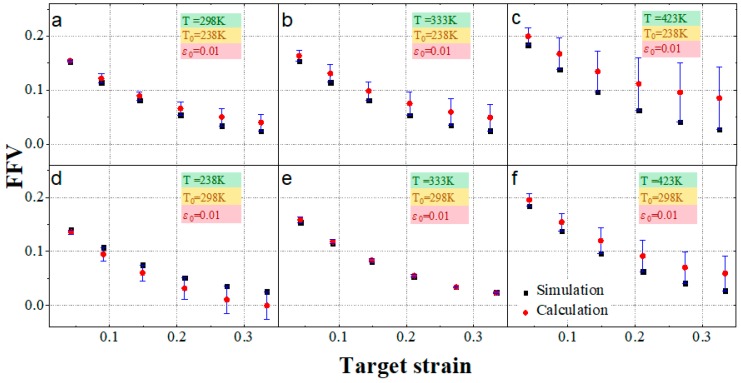
The free volume of the temperature–strain superposition: the black square represents the simulation result, the red circle represents the calculation result of the temperature–strain superposition principle, and the blue vertical line represents the difference between calculation and simulation. At the reference temperature of 238 K and the reference strain of 0.01, (**a**–**c**) are calculations and simulations at target temperatures of 298, 333, and 423 K, respectively. At the reference temperature of 298 K and the reference strain of 0.01, (**d**–**f**) were calculated and simulated at target temperatures of 298, 333, and 423 K, respectively.

Both temperature and strain can affect the viscoelastic performance of EPDM by affecting the fractional free volume. The feasibility of the superposition between the above two factors was verified by comparing the calculated results with the simulated results. The error analysis results of the free volume derived from Equation (8) and the simulation results are shown in Figure 14:

As shown in Figure 14, the variation trend of the superimposed calculation results is consistent with the simulation results. The free volume increases as the target temperature increases, and it decreases as the target strain increases. However, with the exception of Figure 14e, the calculated and simulated results of the free volume in each figure have obvious errors. When the difference between the reference temperature and the target temperature is maximum, the reference strain and the target strain are maximum (Figure 14c, $T_{0}$ = 238 K, T = 423 K, $\varepsilon_{0}$ = 0.01, *ε* = 0.33), with a maximum error of 0.058.

According to Figure 14a,c, under the same reference temperature and reference strain, when the target temperature is higher than the reference temperature, the error gradually increases as the target temperature increases, with the superposition-predicted results higher than the simulated ones. By comparing Figure 14c with Figure 14f, under the same reference strain and target temperature, when the reference temperature is lower than the target temperature, the smaller the difference between them, the smaller the error. By comparing Figure 14d,f, under the same reference temperature and reference strain, when the target temperature is lower than the reference temperature, the superposition calculation result is smaller than the simulation result, and the exact opposite is observed when the target temperature higher than the reference temperature. The error may be acceptable with the appropriate reference temperature. As shown in Figure 14e, when $T_{0}$ = 298 K and *T* = 333 K, the superposition-predicted results are almost consistent with the MD simulation results.

## 4. Conclusions

Cross-linked EPDM was constructed through MD simulations in this study to understand the fundamentals associated with EPDM’s viscoelastic behavior. The MD model of EPDM was verified by comparing it with experimental results for the glass transition temperature, the stress–strain relationship, and the gas diffusion coefficients in the literature. The dependence of FFV on temperature, strain, and their combined effect was investigated; in addition, any equivalent effects among these influencing factors were evaluated, on the basis of which the applicability of TTSP and other superposition principles was also investigated. The main conclusions are drawn as follows:

1. The MD simulation results for the glass transition temperature and stress–strain curve were compared with values in the literature, and there is good agreement. The simulated O2 diffusion coefficient is closer to the experimental value than the Rutherford’s simulation, as the cross-linked EPDM model in the current study is closer to the experimental one.
2. The fractional free volume is proportional to temperature only when the target temperature is lower than 330 K; the temperature expansion coefficient is approximately 0.000239, and while higher than 330 K, the temperature expansion coefficient is no longer constant, and this is consistent with the applicable temperature range of WLF equation, $T_{g}$~$T_{g}$ + 100.
3. The fractional free volume of EPDM decreases exponentially with increasing strain magnitude, which can be expressed as ${f-f}_{0}{\;=\;f}_{0}{(\exp(-25/4(\varepsilon -\varepsilon }_{0}))-1)$.
4. The free volume predicted by temperature–strain superposition has a significant error compared with the MD simulation results. The error is acceptable only when the reference temperature is 298 K and the target temperature is 333 K, so the superposition of temperature and strain magnitude is feasible only in a particular situation.
5. TTSP is applicable for EPDM at temperatures below 330 K; the assumptions of TSSP have proven to be unsuitable for EPDM. The contribution of temperature and strain to free volume can barely be superimposed at the appropriate reference temperature, so the applicability of TTSSP to EPDM is also limited.

## Figures and Tables

**Table 1:** Goodness-of-fit check.

Reference Strain	Reference Fractional Free Volume (*f_0_*)	A	A	B	B	Statistics	Statistics
		Value	Standard Error	Value	Standard Error	Reduced Chi-Sqr	Adj. R-Square
ε_0_ = 0	0.203866	0.2048	0.00311	−0.20862	0.00365	2.14 × 10^−6^	0.99944
ε_0_ = 0.01	0.187926	0.19096	0.0037	−0.19027	0.00375	3.04 × 10^−6^	0.99937
ε_0_ = 0.04	0.153478	0.15704	0.00422	−0.15496	0.00375	3.04 × 10^−6^	0.99937
ε_0_ = 0.09	0.114238	0.11673	0.00453	−0.11623	0.00375	3.04 × 10^−6^	0.99937
ε_0_ = 0.15	0.080902	0.08151	0.00437	−0.08356	0.00375	3.04 × 10^−6^	0.99937
ε_0_ = 0.21	0.052951	0.05548	0.00386	−0.05637	0.00375	3.04 × 10^−6^	0.99937
ε_0_ = 0.27	0.03418	0.03769	0.00323	−0.03622	0.00375	3.04 × 10^−6^	0.99937
ε_0_ = 0.33	0.024388	0.02608	0.00264	−0.02616	0.00375	3.04 × 10^−6^	0.99937
